# Shrub avoidance by an open-adapted ground squirrel in a shrub-encroached environment

**DOI:** 10.1371/journal.pone.0297993

**Published:** 2024-02-12

**Authors:** Alexandra D. Burnett, John L. Koprowski

**Affiliations:** 1 School of Natural Resources and the Environment, University of Arizona, Tucson, AZ, United States of America; 2 Haub School of Environment & Natural Resources, University of Wyoming, Laramie, WY, United States of America; Wrocław University of Environmental and Life Sciences: Uniwersytet Przyrodniczy we Wroclawiu, POLAND

## Abstract

Habitat loss and degradation are leading drivers of the widespread decline in wildlife populations, and understanding how wildlife perceive and navigate their environment is useful for predicting responses to future landscape changes. Small mammals play an important role in their environments, however, many species are threatened by rapid environmental change. The Harris’ antelope squirrel (*Ammospermophilus harrisii*) is endemic to the Sonoran Desert but faces multiple landscape changes due to anthropogenic activity. We fitted *A*. *harrisii* with radio collars to quantify resource selection and better understand how further environmental change may affect squirrels. Squirrels exhibited differential selection depending on behavior and scale. When selecting for microsites suitable for burrows and alarm calling (i.e., fourth-order selection), squirrels selected for both cacti and shrub portions of the habitat. Overall habitat selection within home ranges (i.e., third-order selection) showed selection against shrub patches, however, suggesting that further shrub encroachment may have consequences for *A*. *harrisii* behavior and distribution.

## Introduction

Habitat loss and degradation are leading drivers of extinction [1], decreasing resources available for populations to recover following extreme climate or disturbance events [2, 3]. Arid environments with high temperatures that are prone to drought are particularly threatened by habitat degradation and fragmentation [4, 5], and these threats are further exacerbated by climate change [6]. A number of species at the forefront of these widespread landscape changes are understudied, however, and further research about their ecology is needed to predict how populations may respond to ongoing environmental change.

Small mammals play an integral role in community composition by affecting plant distribution and cover [7–11], soil characteristics [10, 12], and insect abundances [10]. These effects may be more pronounced in desert environments with low resource density [7, 10]. Turnover of small mammal communities is common in response to rapid environmental changes like shrub encroachment [3, 13] and urbanization [14–16], in which generalists are favored over specialists. Loss of specialists may in turn accelerate landscape transitions [17] and push disturbed areas past their tipping point into an alternative stable vegetative state [18–20]. Many small mammal species are cryptic, however, and both research and management of such species may be difficult. With little ecological knowledge and unreliable means of detection, populations of understudied species at the forefront of environmental change may be especially vulnerable to such changes if declines go unnoticed or cannot be readily detected or measured.

Changes in vegetation structure or community can disrupt behaviors necessary for survival or reproduction, particularly for specialists adapted to specific environments [21, 22], and understanding how species select and secure resources is important for predicting how species will respond to environmental change [23, 24]. Resource selection studies measure how resource use compares to resource availability [25, 26], providing insight into how a particular species perceives and responds to their surrounding environment. Resource selection analyses allow researchers and wildlife managers to map animal distributions [27], assess habitat requirements [28, 29], identify movement corridors [30], and direct habitat preservation [31] and restoration [24, 32].

The Harris’ antelope ground squirrel (*Ammospermophilus harrisii)* is a small-bodied (~120g), diurnal animal endemic to the Sonoran Desert that is behaviorally and physiologically adapted to open, arid environments [33–35]. Much of the Sonoran Desert has faced significant disturbance in the past century. In southern Arizona, urban expansion, agriculture, grazing pressure, road development, and shrub encroachment all threaten native vegetation and wildlife. Much of *A*. *harrisii* distribution in southeastern Arizona is scattered across low elevation desert-scrub currently threatened by development [36, 37] and encroachment of native velvet mesquites, *Prosopis velutina* [38, 39]. *Ammospermophilus harrisii* are listed as a “Species of Greatest Conservation Need (SGCN)” in Arizona [40], and the San Joaquin Antelope Squirrel (*Ammospermophilus nelson)*, a congener in the San Joaquin Desert of California, is listed as Threatened by the state of California [41]. To better understand the ecology of *A*. *harrisii* and how antelope squirrels might respond to ongoing environmental change, we studied resource selection and space use in southeastern Arizona.

## Materials and methods

### Study area

The Santa Rita Experimental Range is a 21,000ha research facility in Pima County, Arizona, approximately 50km south of Tucson. The region receives ~300mm of rainfall yearly in a bimodal pattern, driving late seasonal vegetation blooms and peaks in wildlife activity characteristic of desert grasslands [42, 43]. Vegetation composition shifts from open Sonoran desert-scrub at ~900m to savanna woodlands at ~2500m elevation and is largely dominated by transitional desert grasslands [41]. Over the past century, there has been a well-documented increase in velvet mesquite (*P*. *velutina)* at the Santa Rita Experimental Range [43]. Mesquite trees previously constrained to low-elevation riparian areas have spread upwards in elevation and now represent the dominant shrub between 1000–1200m [43].

We focused on a specific region within the Santa Rita Experimental Range covering ~4km^2^ between a 950 and 1150m elevation gradient where *A*. *harrisii* were most abundant, which we hereafter refer to as the study area. The vegetation between these elevations exhibited a largely uniform distribution composed primarily of desert scrub and cacti, including prickly pear (*Opuntia* spp.*)*, mesquite (*Prosopis* spp.), cholla, (*Cylindropuntia* spp.), and desert hackberry (*Celtis ehrenbergiana)*. A dirt road bisects the study area, running from the southern end to the northern end of the study area, which corresponds with a gradient from higher elevation (~1000m), higher density shrub cover to lower elevation (~900m), lower density shrub cover.

### Data collection

We collected data at our study area at least twice weekly year-round from January 2017 through December 2018. We initially used the road as a transect and placed two traps (model No. 201, Tomahawk Live Trap, Hazelhurst, WI, U.S.A) every 300m. Due to low squirrel density, however, we shifted to trapping opportunistically where squirrels were thought to be present, focusing on five ~0.15km^2^ sites across a gradient of shrub densities. We baited traps with peanut butter to humanely capture individuals. All procedures followed guidelines for ethical treatment of animals and obtained approval from the University of Arizona Institutional Animal Care and Use Committee (protocol no. 16–169). We wrapped traps in shade cloth and placed traps in well-shaded locations to avoid heat stress to captured animals. We checked traps every hour when traps were open. We used a cloth handling cone [44] to handle squirrels and recorded weight, sex, life stage, and reproductive status of captured animals (n = 78, 44 females, 34 males).

We fit a VHF radio collar (Wildlife Materials; 3-4g, <5% body weight) to 37 adult *A*. *harrisii* of both sexes (22 females, 15 males). We used a homing strategy [45] to locate individuals at least twice weekly throughout the year, and up to eight times weekly during the summer season (May–August) until collars failed or the individual could no longer be found. We removed collars just before or just after battery failure, placing traps where we had most recently observed the target individual.

During each field visit, we worked throughout the study area to monitor traps or track collared squirrels. We used a Garmin eTrex Legend ® GPS unit to record the geographic coordinates of all visual or acoustic detections (i.e., alarm calls) of *A*. *harrisii* in our study area, including opportunistic detections, capture locations, and telemetry locations. For each geocoordinate in which the squirrel location was confirmed visually, we recorded squirrel behavior (e.g., grooming, digging, etc.), distance from the nearest burrow, distance from cover, and the nearest plant species within approximately 5m. These variables were used to isolate and classify burrow and alarm locations for selection indices pertaining to these microhabitats. Behavioral data included the behavior the squirrel was performing at the time of tracking (e.g., digging, heat dumping, alarm calling, etc.), and any notes specifying the context of the behavior (e.g., whether other individuals were present, whether individual was foraging or maintaining burrow, etc.).We obtained geocoordinates of acoustic detections by following the sound of the antipredator vocalization, approaching slowly until the squirrel was within view, or we were within approximately 15m of the squirrel. If the calling squirrel was successfully located, we recorded the same variables described above, making note of which plant species the squirrel called from, if any. We used vegetation data of call sites to inform selection indices regarding vegetation used for alarm calling. Acoustic detections in which the calling squirrels were not visually detected were not included in our selection analyses.

### Statistical analysis

To inform resource selection models, we loaded high resolution camera imagery of the study area from August 2017 provided by NEON [46] into ArcMap 10.8 (Esri Inc., Redlands CA) and ran a supervised classification analysis [47] to allocate our study area into four classes: bare ground, herbaceous cover, cacti cover, and shrub cover. The bare ground class consists of stretches of sandy or rocky soil between sparse desert vegetation. During the monsoon season (July–September), however, grasses, including bush muhly (*Muhlenbergia porteri*) and Rothrock’s grama (*Bouteloua rothrockii)*, experience a peak in growth and can form a blanket over previously bare ground. Herbaceous cover represents areas that maintain enough moisture to allow taller grasses, such as tanglehead (*Heteropogon contortus*) and Lehmann’s lovegrass (*Eragrostis lehmanniana*), and forbs to persist year-round and are generally confined to riparian zones and higher elevations. Thus, overall vegetation structure remains relatively stable across seasons such that “bare ground” and “herbaceous” categories remain distinct structurally. We did not test for variation in selection based on seasons, an assumption which requires further investigation in the future. Despite imagery taken during the monsoon season, a clear visual difference remained between previously bare ground and herbaceous cover, allowing for classification. Cacti patches were primarily composed of prickly pear and cholla. Shrub patches were primarily composed of velvet mesquite and desert hackberry. Based on maximum likelihood estimates of supervised classification, the entire 4km^2^ study area was composed of approximately 17.7% woody shrubs, 18.6% cacti, 48% bare ground, and 15.6% herbaceous cover. We used supervised classification output to classify used and random locations included in third-order resource selection models. Observations used for fourth-order selection of microhabitat (e.g., burrow and alarm locations) were instead classified based on direct observations of plant species within 1m. All subsequent statistical analyses were performed in R [48] using RStudio [49].

For resource selection analyses, we quantified selection behaviors within home ranges (third-order selection) as well as the use of common plant species for burrows and alarm calling locations within each vegetation class (fourth-order selection) [25]. Resource selection may vary with behavior, and a resource that is selected during one behavior may be avoided during another [50]. We were specifically interested in analyzing microhabitat selection for burrow and alarm locations because of the importance of nesting and alarm calling behaviors for survival and reproduction [51–53].

For third-order selection, we modeled use-availability data consisting of known squirrel locations obtained during tracking and random locations generated within each squirrel’s home range. To generate home ranges, we used the “move” package [54] to determine minimum locations per individual needed before home range size plateaued. Based on these results, we used the “adehabitatHR” package [55] to calculate 95% and 50% kernel densities with more than 20 locations (n = 18 squirrels: 12 females and 6 males). We additionally used ArcMap to calculate Minimum Convex Polygons (MCP) for collared squirrels and generate random points within each home range. We used MCPs to generate random points because home ranges calculated using 95% kernel densities were composed of several patches. Random points projected within 95% kernel densities would therefore be biased toward resources underlying patches and remove our power to identify which variables dictate these patches. We used ArcMap to generate 100 random locations within MCP boundaries of each individual and assigned a vegetation class to each point based on supervised classification output. To avoid including points in which squirrel location was influenced by observer presence, all points in which the squirrel moved just before homing or in which a point had been taken within the prior hour were eliminated from home range analyses and selection models. To test whether home range size varied significantly between sex and season, we used Welch’s t-test to account for unequal variance and sample sizes.

We initially considered vegetation class (e.g., bare ground, shrub, cacti, or herbaceous), distance to the nearest known burrow (i.e., the nearest burrow in which we observed use by *A*. *harrisii* at least once*)*, and minimum distance to a dry riverbed, or wash, which may be used for travel, as potential input variables for our resource selection function. Because we have observed antelope squirrels using several burrows within their home range and because different squirrels may occupy the same burrow (at different times or simultaneously), we calculated the Euclidean distance from each squirrel location to the nearest known burrow using ArcMap. We ran a similar analysis for distance to wash using aerial imagery to map washes. We standardized all distance measurements to use as predictor variables in our models. We also included a standardized variable for squared distance from burrow and squared distance from wash to allow for nonlinear relationships. We used the ‘lme4’ package [56] to test a mixed-effects logistic regression model of a binary response variable consisting of used locations (y = 1) and random locations (y = 0) generated within MCPs.

Though initial analyses indicated that distance to the nearest wash was a significant predictor of squirrel presence, burrow and wash distance showed a significant positive relationship with one another. Because distance to burrow showed greater explanatory power, we removed distance to wash from our final input model, or global model, to avoid inference errors due to collinearity. Wash distance may have important influence over second-order selection, or where squirrels choose to settle. Following methods outlined by Grueber and co-authors [57], we used the *“*MuMIn*”* package [58] to generate a model selection table of all possible model combinations of variables included in the global model and used the Corrected Akaike’s Information Criterion (AIC_C_) corrected for small sample sizes to select our top resource selection models [59]. Our global model included vegetation class, standardized distance to burrow, squared standardized distance to burrow, and a random effect to account for individual ID, such that each individual’s locations were compared only to random locations generated within that individual’s home range. Our table revealed two top models within two ΔAIC_C_, which we averaged together to obtain our final model. Because we were specifically interested in the effect of shrubs on squirrel presence, we took the conditional average of our top models within two ΔAIC_C_, rather than using the zero method (i.e., full average) and risking shrinkage toward zero [52]. One of two top models did not show vegetation class as a significant predictor, therefore, taking the full average of both models would include a “0” beta value for vegetation class and potentially underestimate the effect of shrubs on squirrel presence.

For fourth-order analysis, we used distance from cover and plant species recorded within 1m to classify burrow and alarm locations into the four vegetation classes. Burrows or alarm locations that were greater than 1m from cover were classified as the “bare ground” vegetation class. To quantify the strength and direction of fourth-order selection of burrows and alarm calling locations, we calculated Strauss’ [60] selection index (proportion used–proportion available) to compare the proportions of direct observations within each vegetation class to those of random locations generated within the home ranges of all individuals. Values falling above one indicate positive selection and values falling below one indicate avoidance. We estimated significance of observed patterns with Pearson’s chi-squared test. We included observations of both marked and unmarked individuals in selection analyses of alarm calling locations. We pooled individuals together and compared our observations against random locations across all individuals because home ranges of observed squirrels were not always available (e.g., if the squirrel was uncollared or not enough points were obtained for accurate home range calculation).

### Ethics statement

This study was conducted under approval of the University of Arizona Institutional Animal Care and Use Committee (protocol #16–169). All field research was conducted under appropriate state (AZGFD LIC #SP501610 and AZGFD LIC #SP611944) and local (Santa Rita Experimental Range, RUA 16–134) permits. All procedures followed established guidelines for ethical treatment of animals.

## Results

We captured 78 squirrels (44 females, 34 males) over the two-year project, including 7 juveniles (5 females, 2 males). We fitted 37 adult squirrels with radio collars (22 females, 15 males). Of these individuals, we were able to collect enough points to calculate a home range for 18 individuals (12 females, 6 males). Home ranges averaged (±SE) 2.1± 0.2ha (100% MCP) and 2.5± 0.5ha (95% kernel densities) with a 0.5±0.1ha average 50% core area (n = 18 squirrels). Female home ranges (95% kernel densities; 2.6±0.6ha) were slightly larger than male home ranges (2.1±0.9ha), however this difference was not significant for either 95% ranges (Welch’s t-test; t = -0.5, df = 10.7, p = 0.6) or 50% core ranges (t = -0.4, df = 8.4, p = 0.7). Home ranges of *A*. *harrisii* varied considerably by individual, however, ranging from approximately 0.5ha to 7ha. Antelope squirrels exhibited overlap of female-female, female-male, and male-male home ranges, however, squirrels often rotated activity around core areas within their home range, and we did not have a high enough temporal resolution of points to calculate percentage overlap while accounting for spatiotemporal shifts within home ranges. Home ranges also varied by season, expanding during cooler months and contracting during warmer months. Individuals tracked October through March exhibited home ranges averaging 3.6±0.6ha (n = 4 individuals), whereas individuals tracked April through September exhibited home ranges averaging 2.6±0.6ha (n = 12 individuals). However, these differences were not significant for 95% home ranges (Welch’s t-test; t = 1.3, df = 10.1, p = 0.2) or 50% home ranges (t = 1.6, df = 8.3, p = 0.2).

**Fig 1:**
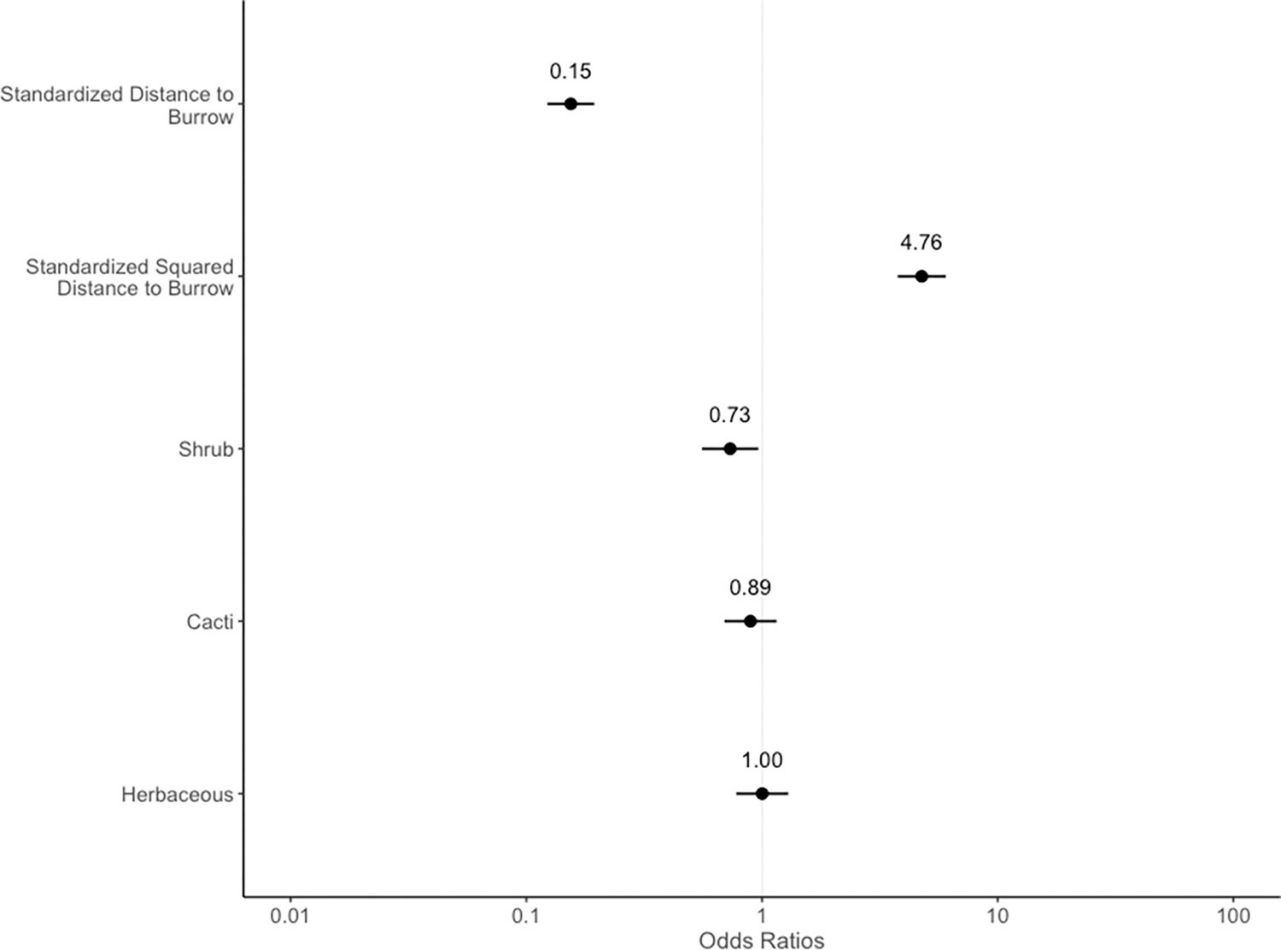
Coefficients of our top model for third-order resource selection. Back-transformed model coefficients showing the effects of burrow distance and vegetation class on the odds of squirrel presence. The odds of squirrel presence were highest at intermediate distances to burrows and decreased in shrub and cacti patches. Coefficients are based on the conditional average of our top two models. Bare ground was used as the reference class because ~50% of the study area was comprised of this vegetation class (see Methods).

Model selection based on AIC resulted in two top models of use-availability data with variables including class, distance to burrow, and squared distance to burrow. Distance to burrow (ß_1_ = -1.9, SE = 0.1) and squared distance to burrow were significant predictors of squirrel presence (n = 18 squirrels, ß_2_ = 1.6, SE = 0.1). The probability of squirrel presence decreased as distance to known burrows increased, but this effect reversed at larger distances, indicating that squirrels have the highest probability of being found at intermediate distances. Vegetation class coefficients showed that squirrels did not strongly select for or against herbaceous cover in relation to bare ground (ß = 0.002, SE = 0.1), but selected against cacti (ß = -0.1, SE = 0.1) and shrubs (ß = -0.3, SE = 0.1; Fig 1) in relation to bare ground.

**Fig 2:**
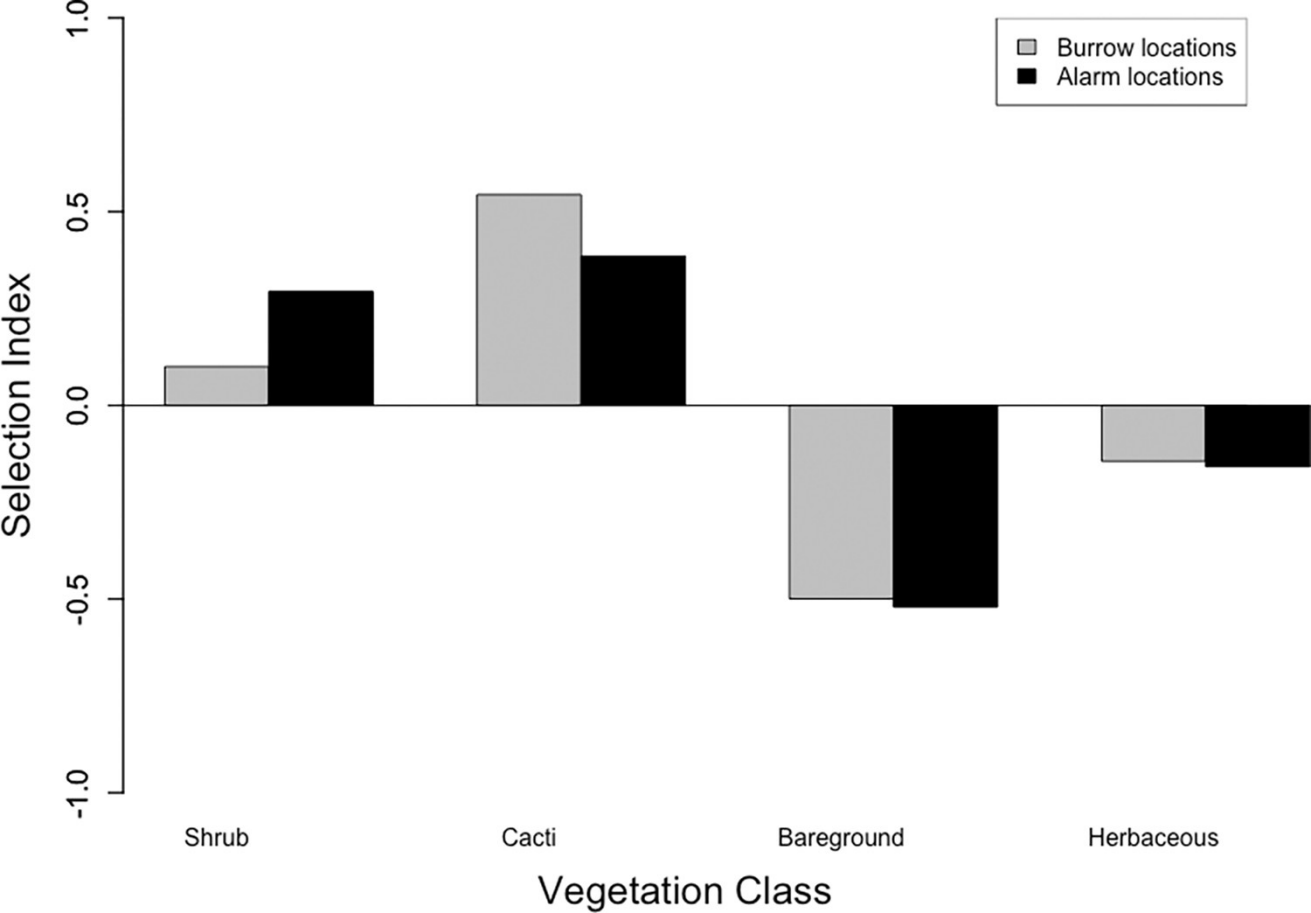
Fourth-order selection indices of burrow locations and alarm locations of *Ammospermophilus harrisii* in southeastern Arizona. Selection of burrow locations (gray) and alarm locations (black) within each vegetation class based on direct observations compared to availability across study sites. We included all burrow locations that were used by marked individuals at least once (n = 29 animals, 142 total burrow locations). For alarm locations, our analyses included only observations in which the plant the individual was calling from was positively identified (n = 33 observations). Negative values indicate selection against the vegetation class, positive values indicate selection for the vegetation class. Burrows beneath *Opuntia* were used most commonly. *Prosopis* and *Celtis* were the next most frequent genera for burrowing beneath, though at a lower frequency. Individuals also used burrows beneath *Senegalia* and *Cylindropuntia*, as well as *Ferocactus*, *Ephedra*, and *Heteropogon*. Individuals called most frequently from *Opuntia* and *Prosopis* but also used *Celtis*, *Cylindropuntia*, and *Ferocactus*.

Squirrels dug burrows most frequently beneath prickly pear (*Opuntia* spp., 66% of observations) but also occasionally used woody shrubs such as mesquite (*Prosopis* spp., 9% of observations), desert hackberry (*Celtis ehrenbergiana;* 8%) and catclaw acacia (*Senegalia greggii;* 6%; n = 29 animals, 142 observations). When choosing burrow sites, squirrels selected for cacti and shrubs and selected against bare ground and herbaceous cover compared to expected values assuming neutral selection toward vegetation classes (χ^2^ = 357.5, df = 3, p<0.001; Fig 2). When selecting for specific alarm calling sites, squirrels again selected for both cacti and shrub classes and selected against bare ground and herbaceous cover (n = 33 observations, χ ^2^ = 70.7, df = 3, p<0.001; Fig 2). Individuals were seen alarm calling in prickly pear and mesquite in 45% and 30% of known calling locations, respectively. We also observed squirrels alarm calling from desert hackberry (*Celtis ehrenbergiana*, 15%), cholla (*Cylindropuntia* spp., 6%), and fishhook barrel cactus (*Ferocactus wislizeni*; 3%).

## Discussion

*Ammospermophilus harrisii* exhibited home ranges smaller than reported for other antelope squirrels [61–63], but larger than many other North American ground squirrels [64–69]. For example, *A*. *nelsoni* average approximately 5ha [61], whereas, round-tailed ground squirrels (*Xerospermophilus tereticaudus)*, which are sympatric with *A*. *harrisii*, have a home range of only 0.3ha [64]. Many other North American ground squirrels also exhibit small home ranges, including Richardson’s ground squirrel [65], Townsend ground squirrels [66], thirteen-lined ground squirrels [67], California ground squirrels [68], and golden-mantled ground squirrels [69], which all exhibit home ranges at or below approximately 1ha. Like other antelope squirrels, home ranges of *A*. *harrisii* were variable, ranging from approximately 0.5ha to 7ha. In comparison, home ranges of white-tailed antelope squirrels (*Ammospermophilus leucurus)* ranged from 3-8ha [62]. *Ammospermophilus harrisii* exhibited behavior very similar to that described by Bradley [62] regarding *A*. *leucurus*, in which individuals used a smaller “daily range” encompassed within their general home range. These “daily ranges” were fluid from day to day and shifted gradually with time. Like *A*. *leucurus*, *A*. *harrisii* also used multiple burrows throughout their home range, and while burrows were often used a few to several days in a row, squirrels regularly moved to a different burrow. We did not find significant seasonal or sex differences in home range size. Low sample sizes for each group could be limiting our power to discern seasonal or sex differences, and future studies are needed to better understand how ecological or physiological factors may drive variation in space use by *A*. *harrisii*.

Our results indicate that our study population of *A*. *harrisii* uses cacti and shrubs as a resource for alarm calling and burrow locations but also may depend on open conditions to avoid predation while foraging. Squirrels exhibited positive selection for cacti and shrubs for burrowing and antipredatory behavior but selected against shrub patches. Distance from burrow was a significant predictor of squirrel presence, suggesting that either the burrows themselves or the vegetation associated with burrows are highly valuable resources. Burrows are extremely important for antelope squirrels to dissipate heat and maintain diurnal activity through extreme temperatures [70]. Squirrels most often burrowed beneath prickly pears, which offer food, shelter from the heat, a high vantage point to detect predators approaching, and cover from predators when they arrive. Although squirrels selected for shrubs when alarm calling or burrowing, third-order selection analyses show that squirrels select against shrub patches in their home range. Thus, while mesquite trees could serve as an important resource, squirrels may rely on open environments to maintain visibility while foraging. Further shrub encroachment could result in local extirpation of antelope squirrels in heavily encroached areas or a shift in the distribution of *A*. *harrisii* across the landscape if dispersing individuals select against areas with high shrub density.

Avoidance of shrub patches is commonly observed in species adapted to open environments, including other ground squirrels [71, 72], nocturnal rodents [13], as well as large African herbivores [20]. For example, habitat use in long-tailed ground squirrels (*Spermophilus undulatus)*, a ground squirrel living in steppe environments, is similarly dependent on bush density, and *S*. *undulatus* does not tolerate shrubs at densities above 35% [71]. Likewise, in the San Joaquin Desert, where the invasion of exotic grass species has resulted in higher vegetation cover than historically known, the abundance of several endemic vertebrates, including *A*. *nelsoni*, was negatively associated with increasing herbaceous biomass, and cattle grazing resulted in increases in abundances [73]. Similar to our results, although *A*. *nelsoni* are strongly associated with saltbush, *A*. *nelsoni* were found more often on sites with sparse or medium shrub cover [74] and have been observed at equal or greater densities in shrub-less environments [75]. Dense shrub cover presents challenges for maintaining visibility for predator evasion and may require a higher state of vigilance while foraging, decreasing foraging efficiency [13, 72, 76]. *Ammospermophilus harrisii* and other open-adapted species may therefore face steep trade-offs as shrub density increases despite its usefulness as a resource.

Our findings provide evidence of potential consequences for the distribution of *A*. *harrisii* resulting from habitat degradation. Like other small mammals, *A*. *harrisii* may play an important role as seed dispersers. We frequently observed *A*. *harrisii* carrying fruits and seed pods and occasionally bringing food items into the burrow. This foraging behavior was found to be extremely important for plant distribution in *A*. *harrisii’s* congener, *A*. *leucurus* [8]. Antelope squirrels likely benefits the Sonoran Desert vegetation in a manner similar to that of *A*. *leucurus* and other ground squirrels, and changes in the spatial distribution of *A*. *harrisii* could have cascading consequences for Sonoran vegetation. Close behavioral monitoring of *A*. *harrisii* and other vulnerable and/or understudied species will be instrumental for identifying consequential or maladaptive responses to landscape transitions. Ideally, habitat connectivity should be preserved where shrub encroachment and other habitat disturbances are a concern to reduce the risk of population fragmentation and local extirpation [77, 78].
